# Beneficial Effects of Alternate Dietary Regimen on Liver Inflammation, Atherosclerosis and Renal Activation

**DOI:** 10.1371/journal.pone.0018432

**Published:** 2011-03-31

**Authors:** Peter Y. Wielinga, Gopala K. Yakala, Peter Heeringa, Robert Kleemann, Teake Kooistra

**Affiliations:** 1 TNO-Metabolic Health Research, Leiden, The Netherlands; 2 Medical Biology Section, Department of Pathology and Medical Biology, University Medical Center Groningen, University of Groningen, Groningen, The Netherlands; 3 Top Institute Food and Nutrition, Wageningen, The Netherlands; Maastricht University, Netherlands

## Abstract

**Background:**

Alternate day calorie restriction (CR) has been shown to be almost as beneficial as daily CR. The question arises whether this concept is also applicable to alternating dietary composition.

**Objective:**

To seek evidence that alternating high cholesterol (HC) - cholesterol-free (CON) Western diet can effectively diminish hepatic and renal inflammation and cardiovascular risk factors as compared with daily HC-supplemented Western diet.

**Design:**

Four groups of ApoE*3Leiden mice, a humanized model for atherosclerosis, were subjected to different feeding treatments for 16 weeks. Mice were fed CON diet; CON diet with 1% w/w cholesterol (HC); alternate (ALT) diet regimen of CON (4 days) and HC (3 days); or CON diet supplemented with 0.43% (w/w) cholesterol (MC), with overall dietary cholesterol intake equal to ALT. Plasma was analyzed for cardiovascular risk factors, aorta for atherosclerotic lesion formation, and liver and kidney for inflammation.

**Results:**

ALT diet but not MC was almost as effective as daily CON feeding in preventing disease development. Compared to HC, the ALT group showed 62% lower hepatic nuclear factor kappa B (NF-κB) activity (P<0.001), a reduction of the circulating inflammatory markers E-selectin (−20%; P<0.05), vascular cell adhesion molecule 1 (VCAM-1; −15%; P<0.05) and Serum Amyloid A (SAA; −31%; P<0.05), smaller atherosclerotic lesion sizes (−51%; 46497±10791 µm^2^ vs. 94664±16470 µm^2^; P<0.05) and diminished renal expression of specific inflammation and activation markers (VCAM-1, −27%; P<0.05; monocyte chemotactic protein-1 (MCP-1); −37%; P<0.01).

**Conclusion:**

Alternate HC-CON feeding reproduced most of the beneficial effects of daily cholesterol-free diet, including strongly diminished hepatic, vascular and renal activation and inflammation; also atherosclerosis was reduced by half as compared to HC, albeit still higher compared to the CON group.

## Introduction

Cardiovascular disease (CVD) is the leading cause of morbidity and mortality in industrialized countries. A better understanding of modifiable risk factors for CVD is critical in order to effectively prevent or retard this disease. Among the modifiable risk factors are unhealthy diet and excessive calorie intake. It is well known that improvements in dietary composition can strongly influence the risk of developing CVD [1]. Similarly, caloric restriction reduces metabolic risk factors for CVD [2]; [3]. However, compliance with these broad guidelines is problematical and not acceptable to most people despite the undisputed beneficial health outcomes. Therefore, novel approaches for dietary interventions should be considered.

It may be more feasible in practice to introduce a form of alternating dietary manipulation than daily dietary changes. In this case dietary changes could be instituted for limited time periods. Intuitively it seems likely that persons will find it easier to adapt to this than to restrict their intake every day. Recent findings in humans suggest that alternate-day fasting may indeed be a viable diet option to help obese individuals lose weight and decrease CVD [4]. Here we wish to seek evidence that alternate dietary composition will also beneficially affect metabolic health. To this end, we evaluated the effect of alternating high cholesterol (HC) diet and cholesterol-free control (CON) diet on liver health and macro- and microvascular function in a humanized mouse model of CVD, the ApoE*3Leiden (ApoE3L) mouse [5].

We previously showed that feeding ApoE3L mice a Western diet supplemented with HC (1% w/w), but not CON diet, induces CVD risk factors such as hypercholesterolemia, systemic inflammation and endothelial dysfunction, which precede atherosclerotic lesion formation [6]. Notably, the marked systemic inflammatory response was shown to be related to liver inflammation. Here we wish to explore whether in a similar experimental set-up an alternate HC diet (3 days) - CON diet (4 days) could diminish hepatic inflammation and CVD risk factors and thereby decrease atherosclerotic lesion formation as compared to continuous HC feeding. To evaluate whether an alternate diet regimen may also be beneficial for the microvasculature, we extended our analyses to the kidney. The alternate diet regimen was also compared to feeding mice daily 0.43% w/w cholesterol (MC), whereby the overall cholesterol intake of the MC group equals that of the alternate group. Our findings indicate that the principle of alternate feeding regimens effectively diminishes metabolic and vascular risk factors and improves the hepatic, renal, and vascular health status in ApoE3L mice.

## Methods

### Ethics statement

The experiment was approved by and is in compliance with the regulations set forward by the Ethical Committee on Animal Care and Experimentation (Zeist, The Netherlands), approval number DEC2688.

### Animals

Sixty female ApoE*3Leiden mice (12–14 weeks old) were group housed (3–5 mice per cage) with a 12 h light-dark cycle (7 a.m.–7 p.m. lights on). The mice had free access to food and water and remained on maintenance chow (Sniff R/M diet V1530, Uden, The Netherlands); composition specified in [7]) until the start of the study. At T = 0, the start of the study, the mice were divided into four groups matched for plasma cholesterol and experimental diet treatment was started. The first group (control group; “CON”) received an established [8] cholesterol-free Western type diet (CON diet; rodent diet T, Hope Farms, Woerden, The Netherlands). The second group was fed the same diet as CON but supplemented with a high dose (1.0% w/w) of cholesterol (high cholesterol group; “HC”). The third group was subject to alternate diet feeding, i.e. 3 days feeding of HC diet followed by 4 days of cholesterol-free CON diet (alternating cholesterol group; “ALT”). The fourth group was treated with the same diet as CON but supplemented with 0.43% w/w of cholesterol (medium cholesterol group; “MC”) to achieve the same dietary cholesterol intake as with ALT. The dietary regimens are illustrated in Figure S1. Body weight (individually) and food intake (at cage level) were monitored over time and blood samples were taken by tail incision after 4 h of fasting (9 a.m. – 1 p.m.) at T = 0 and on day 24, 52, 80 and 108, i.e. always at the end of a 3-days HC feeding period in the ALT group. To further analyze the effect of alternate cholesterol feeding on predicted fluctuations of plasma cholesterol, additional plasma was collected in the ALT group at T = 28, 56, 84 and 112 days (i.e. the last day of the CON feeding periods) and at T = 31, 59, 87, 115 days (i.e. the last day of the HC feeding periods) (Figure S1). After sixteen weeks of experimental diet feeding, the mice were sacrificed by CO_2_ and organs were isolated and weighed. Hearts and kidneys (*sinistra*) were fixed and embedded in paraffin; livers and kidneys (*dextra*) were snap frozen in liquid N_2_ and stored at −80°C until further use.

### Plasma analyses

Total plasma cholesterol and triglyceride levels were measured using kits No. 11489437 and 11488872 (Roche Diagnostics, Almere, The Netherlands), respectively, and the plasma levels of soluble vascular cell adhesion molecule 1 (sVCAM-1; R&D Systems), E-selectin (R&D Systems) and Serum Amyloid A (SAA; Biosource) were determined by ELISA [9].

### Liver inflammation

Nuclear factor kappa B (NF-κB) activity in liver was determined as described [10], using TransAM transcription factor assay kit no. 40097 (Active Motif Europe, Rixensart, Belgium). Briefly, liver homogenates were prepared using the Nuclear Extract Kit (no. 40010, Active Motif, Rixensart, Belgium). Equal amounts of protein (6 µg/well) of the liver homogenates were used to determine the amount of active p65-NF-κB.

### Free cholesterol and cholesterol ester in liver

To determine intrahepatic cholesterol concentrations, liver samples were homogenized. Cholesteryl acetate (2 µg) was added to each sample as an internal standard. Lipids were extracted according to the ‘Bligh and Dyer method’ and neutral lipids were separated by high performance thin layer chromatography on silica-gel-60 pre-coated plates. Quantification of the lipid amounts was performed by scanning the plates with a Hewlett Packard Scanjet 4500c and by integrating the density areas with Tina version 2.09 software.

### Atherosclerotic lesion analysis

Serial cross sections (5 µm-thick) were taken throughout the entire aortic root area for histological analysis of atherosclerosis as described [11]. Briefly, paraffin-embedded aortic cross-sections were stained with hematoxylin-phloxine-saffron and atherosclerotic lesion area was analyzed blindly in 4 cross-sections of each specimen (at intervals of 50 µm). Cell-D software (Olympus Soft Imagine Solutions GmbH) was used for morphometric computer-assisted quantification of lesion number, lesion area and lesion severity according to the classification of the American Heart Association as established [12]. MAC-3 (BD-Pharmingen, cat# 550292) antibody was used to determine macrophage content. MAC-3 positive staining of the atherosclerotic lesions was quantified using Cell-D software (Olympus Soft Imagine Solutions GmbH). CON mice hardly developed atherosclerotic lesions (0.1±0.1 lesions per cross-section) and were therefore not included in further statistical atherosclerosis analysis.

### Renal RNA extraction and gene expression analysis

Total RNA was extracted from thirty 5- µm thin cryo-sections from kidney using RNeasy Mini Plus Kit (Qiagen, Westburg, Leusden, The Netherlands) according to the manufacturer's instructions. Integrity of RNA was determined by Agarose gel electrophoresis. RNA quantity (OD-260) and quality (OD-260/OD-280) were determined using a ND-1,000 UV-Vis spectrophotometer (NanoDrop Technologies, Rockland, DE).

Total RNA was reverse-transcribed using SuperScript III reverse transcriptase (Invitrogen, Breda, The Netherlands) and random hexamer primers (Promega, Leiden, The Netherlands). To detect the expression of selected target genes Assays-On-Demand™ gene expression primer/probe sets (ABI Systems, Foster City, CA) were used. Endogenous PPIA (assay ID Mm02342430_g1) was used as a housekeeping gene along with the following probes: CD68 (assay IDMm00839636_g1), monocyte chemotactic protein-1 (MCP-1; assay IDMm00441242_m1), VCAM-1 (assay IDMm00449197_m1), inter-cellular adhesion molecule 1 (ICAM-1; assay IDMm00516023_m1, P-selectin (assay ID Mm00441295_m1), and E-selectin (assay IDMm00441278_m1). Real-time PCR was performed in duplicate and the obtained threshold cycle (CT) values were averaged. Relative mRNA levels were calculated as 2^-ΔCT^, in which ΔCT is CT_gene of interest_ – CT_PPIA_.

### Renal light microscopy and immunohistochemistry

For light microscopy, 3 µM paraffin sections were stained with Periodic acid-Schiff's (PAS). In short, paraffin sections were deparaffinized and re-hydrated to distilled water. Sections were placed in 0.5% periodic acid solution for 5 minutes. After rinsing in distilled water, sections were incubated in Schiff reagent (Sigma) for 15 minutes, followed by rinsing in lukewarm water for 5 minutes. Sections were counterstained with Mayer's hematoxylin for 1 minute and washed in tap water. Immunohistochemical staining for macrophages and VCAM-1 was performed on acetone-fixed 5 µm cryosections using an anti-rabbit peroxidase-based Envision®+ system (DakoCytomation, Carpinteria, CA, USA). Briefly, sections were incubated for 60 min with rat-anti-mouse CD68 (clone FA11, hybridoma supernatant), VCAM-1 (clone M/K-2.7, hybridoma supernatant) or isotype control antibody (IgG2a, clone OX35, hybridoma supernatant) followed by a 30 min incubation with 10 µg/ml unlabeled rabbit-anti-rat secondary antibody (Vector Laboratories, Burlingame, CA, USA). After detection of peroxidase activity with 3-amino-9-ethylcarbazole, sections were counterstained with Mayer's hematoxylin. Images were taken with a Leica microscope using QwinV3 software, and the extent of macrophage infiltration was determined by morphometry in a blinded manner using Leica Qwin V3 quips (Qwin V3 software, Leica Microsystems Imaging Solutions, Ltd., Cambridge, UK) and expressed as percentage of positive area.

### Statistical analysis

Data were analyzed with SPSS 17.0 for Windows. Changes over time were measured with repeated measures ANOVA with factors treatment and time followed by least significant difference (LSD) post-hoc analysis. Differences between groups at one specific time point were analyzed with 1-way ANOVA followed by LSD post-hoc analysis. Data that were not normally distributed were analyzed with a non-parametric Mann-Whitney U test or a Kruskal-Wallis test followed by a Dunn's test. P<0.05 was considered significant. Results are shown as mean ± SEM.

## Results

### Alternate dietary regimen improves plasma lipids

**Figure 1:**
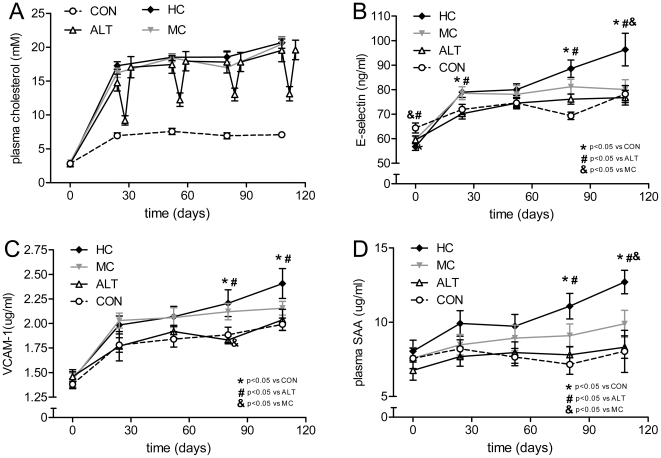
Effect of alternate diet regimen on plasma lipids and inflammation markers. Plasma parameters over time for each treatment group. A) Plasma cholesterol over time, B) plasma E-selectin, C) plasma VCAM-1, D) plasma serum amyloid A (SAA). Groups are abbreviated as: control mice fed a cholesterol-free Western type diet (CON, n = 15); mice fed the same Western type diet but supplemented with high dose (1% w/w) cholesterol (HC, n = 13); mice with alternate CON (4 days) and HC (3 days) diet regimen (ALT, n = 13); mice fed Western type diet supplemented with a medium dose (0.43% w/w) cholesterol (MC, n = 14). * P<0.05 compared to CON, # P<0.05 compared to ALT, and P<0.05 compared to MC. Values are presented as means ± SEM.

Body weight and average food intake did not differ significantly between the groups (Table 1). Starting baseline cholesterol levels at T = 0 were comparable between the groups (2.8±0.1 mM) (Figure 1A). Feeding of a cholesterol-free Western type diet (CON group) resulted in a moderate increase in plasma cholesterol averaging 7.0±2.0 mM. A more pronounced increase in plasma cholesterol was seen with high dose (1% w/w) cholesterol feeding (HC group): after 24 days, plasma cholesterol levels had reached 17.3±0.6 mM, and stabilized at this level for the remainder of the treatment period (average cholesterol 19.4±0.7 mM). Plasma cholesterol in the ALT group fluctuated parallel to the alternate HC diet-Con diet; after periods of HC diet, the plasma cholesterol levels were comparable to those in the HC group, but levels dropped rapidly to on average 11.9±0.9 mM after periods of CON diet, pointing to a rapid adaptation to dietary cholesterol intake. Plasma cholesterol levels in the medium dose (0.43% w/w) cholesterol (MC) group stabilized at 18.0±0.8 mM, slightly lower than those in the HC group.

**Table 1:** Characteristics of the four treatment groups at the end of the study.

	CON (n = 15)	HC (n = 13)	ALT (n = 13)	MC (n = 14)
Body weight (g)	22.8±0.5	23.7±0.4	22.6±0.4	23.8±0.5
Food intake (g/day)	2.45±0.04	2.51±0.07	2.26±0.07	2.29±0.03
Liver weight (g)	1.12±0.05^a^	1.51±0.06^b^	1.11±0.03^a^	1.30±0.04^c^
Intrahepatic cholesterolester (CE) (µg/mg protein)	15.6±0.9^a^	32.2±0.8^b^	28.0±0.6^c^	29.9±.07^c^
Intrahepatic triglycerides (µg/mg protein)	62.9±5.5	76.9±3.5	69.0±2.5	68.7±4.1

Groups are abbreviated as: control mice fed a cholesterol-free Western type diet (CON); mice fed the same Western type diet but supplemented with high dose (1% w/w) cholesterol (HC); mice with alternate CON (4 days) and HC (3 days) diet regimen (ALT); mice fed Western type diet supplemented with a medium dose (0.43% w/w) cholesterol (MC). Data are expressed as means ± SEM. Values in the same row with different superscript letters are significantly different. P<0.05

Plasma triglycerides did not differ between the groups and remained stable at 2.3 mM during the experimental period (data not shown).

### Alternate dietary regimen improves vascular and systemic inflammation

Plasma levels of the vascular inflammation marker E-selectin were on average 60.3±1.6 ng/mL at T = 0 (Figure 1B). Levels in the CON group slightly increased during the study, reaching 78.2±3.5 ng/mL at the end. HC feeding caused a more pronounced and significant (P<0.05) increase in E-selectin with endpoint levels of 96.3±6.7 ng/mL. Notably, alternate diet feeding resulted in E-selectin levels comparable to those of the CON group (74.4±2.4 ng/mL), and significantly lower than the E-selectin levels in the HC group (P<0.01) on days 24, 80 and 108. The MC group showed E-selectin levels slightly, but significantly higher than those in the ALT and CON groups, but lower than in the HC group. For example, on day 24, E-selectin level was 78.5±2.6 ng/mL in MC which was significantly higher than the corresponding values in the ALT (70.3±2.2 ng/mL; P<0.05) and CON (71.9±2.1 ng/mL; P<0.05) groups.

Plasma levels of another vascular inflammation marker, VCAM-1, showed a similar response to the differing feeding regimens as seen for E-selectin (Figure 1C). Levels in the CON group increased from on average 1.38±0.05 µg/mL at T = 0 to 1.99±0.06 µg/mL at the end of the experimental period. HC diet feeding showed a significantly stronger increase in VCAM-1 levels over time, reaching an average endpoint value of 2.41±0.15 µg/mL. Similarly as seen with E-selectin, VCAM-1 levels of the ALT group did not significantly differ from the CON group (endpoint value 2.04±0.11 µg/mL; P = 0.06 compared to HC), while the MC group showed VCAM-1 levels in between the CON/ALT groups and the HC group (endpoint value for MC: 2.16±0.07 µg/mL.

Figure 1D shows the effects of the different diets on the plasma levels of the systemic inflammation marker, Serum Amyloid A (SAA). At T = 0, average plasma SAA levels were 7.4±2.5 µg/mL and remained at this level with the CON diet (endpoint value of 8.0±4.5 µg/mL). Upon HC feeding, plasma SAA levels increased gradually, eventually reaching 12.7±2.7 µg/mL (P<0.01). Again, the alternate diet did not enhance plasma levels (8.4±2.6 µg/mL at the end of the study) and plasma SAA values in the ALT group were very comparable to those in the CON group. Endpoint SAA levels in the MC group (9.9±3.1 µg/mL) were in between the CON/ALT groups and the HC group.

### Alternate dietary regimen reduces liver inflammation

**Figure 2:**
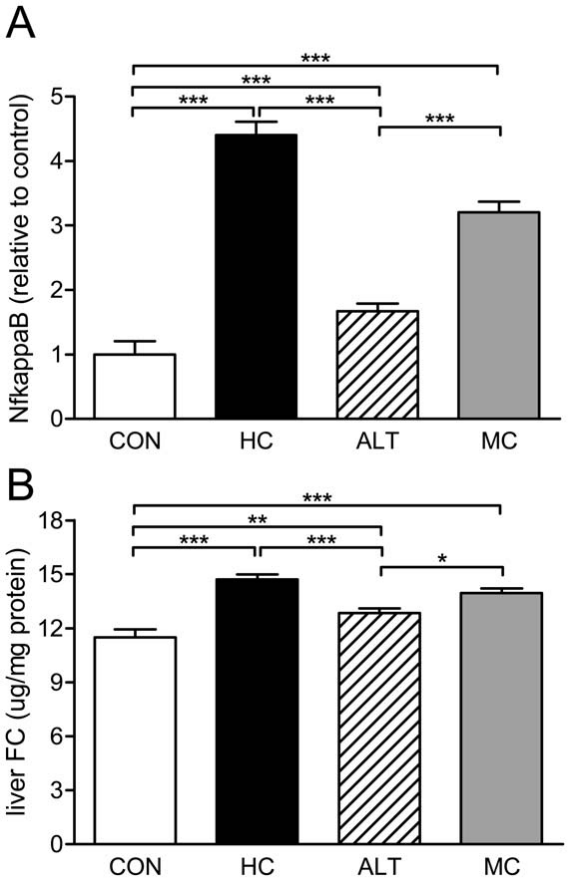
Effect of alternate diet regimen on NFκB activity and intrahepatic lipids. A) Active p65-NFκB expression in liver homogenates relative to CON, B) Intrahepatic free cholesterol concentrations. Control mice fed a cholesterol-free Western type diet (CON, n = 15); high dose (1% w/w) cholesterol diet (HC, n = 13); alternate CON (4 days) and HC (3 days) diet regimen (ALT, n = 13); medium dose (0.43% w/w) cholesterol diet (MC, n = 14). Values are presented as means ± SEM. * P<0.05, ** P<0.01, *** P<0.001.

Since SAA is a liver-derived inflammation marker the expression of which is under control of the inflammatory transcription factor NF-κB, we next compared hepatic NF-κB activity between the groups using liver homogenates prepared at sacrifice. HC feeding was associated with a marked 4-fold (P<0.05) increase in hepatic NF-κB activity (Figure 2A) relative to CON. In contrast, alternating cholesterol feeding hardly affected NF-κB activity and the ALT group displayed a comparable activity as the CON group. A considerable 3-fold (P<0.05) elevation in NF-κB activity was seen with MC. Together, this demonstrates that chronic dietary cholesterol exposure clearly is adverse to the liver and that alternating cholesterol feeding is a suitable way to prevent such effects.

We next analyzed the hepatic concentration of free cholesterol (FC), which is a critical determinant of liver inflammation [13]. Indeed, FC concentrations were significantly higher in the HC (14.7±0.3 µg/mg protein) and MC (14.0±0.3 µg/mg protein) groups as compared to the CON group (11.5±0.4 µg/mg protein; P<0.001) or the ALT group (12.9±0.3 µg/mg protein; P<0.001) (Figure 2B). Similar differences between the groups were found for the concentration of hepatic cholesterol ester (CE) (Table 1). Consistent with the plasma triglyceride data, hepatic triglyceride concentrations were comparable between the groups (Table 1).

### Alternate dietary regimen improves macrovascular function in aorta

**Figure 3:**
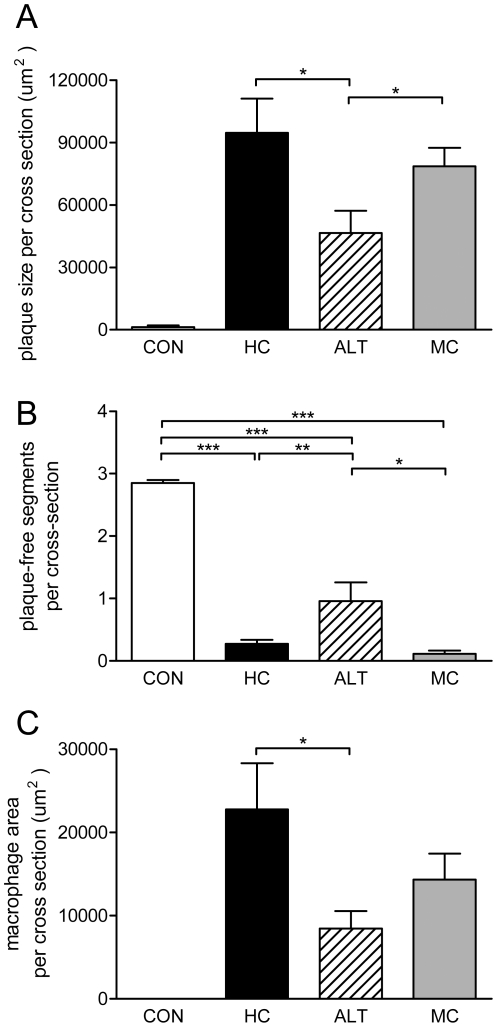
Effect of alternate diet regimen on macrovascular atherosclerotic disease. A) Total atherosclerotic lesion area per cross-section, B) Lesion-free aortic segments per cross section, C) Macrophage immunostaining (MAC-3 immunoreactivity). Control mice fed a cholesterol-free Western type diet (CON, n = 14); high dose (1% w/w) cholesterol diet (HC, n = 12); alternate CON (4 days) and HC (3 days) diet regimen (ALT, n = 12); medium dose (0.43% w/w) cholesterol diet (MC, n = 12). Values are presented as means ± SEM.* P<0.05, ** P<0.01, *** P<0.001.

Atherosclerosis was analyzed after 16 weeks of experimental diet treatment in the aortic valve area of the heart (see Figure S2 for representative photomicrographs). HC feeding and MC feeding resulted in substantial atherosclerosis with total lesion areas of 94664±16470 µm^2^ and 78670±8877 µm^2^ per cross section, respectively (Figure 3A). The total lesion area of the ALT group (46497±10791 µm2 per cross section) was considerably smaller than that of the HC group (−51%; P<0.05) and the MC group (−41%; P<0.05), but still larger than that of the CON group (1305±831 µm^2^). The number of lesions in the ALT and MC groups did not significantly differ from the HC group (on average 3.4±0.3 lesions per cross section; data not shown). Notably, the number of aortic segments free of lesions were significantly higher in the ALT group (0.96±0.30) than in HC (0.27±0.06; P<0.05) and MC (0.11±0.05; P<0.01) (Figure 3B).

### Alternate dietary regimen reduces macrophage content of aortic lesions

While CON livers hardly showed MAC-3 immunoreactivity (IR; Figure 3C), HC treated animals showed pronounced staining with a macrophage-positive area of 22753±5555 µm^2^. Alternate dietary regimen significantly reduced the macrophage-positive IR (8442±2115 µm^2^; P<0.05) relative to HC. MC treatment reduced the macrophage to a lesser and insignificant extent (14341±3124 µm^2^).

Together, the data indicate that alternate cholesterol feeding as compared to continuous cholesterol feeding is beneficial for aortic (macrovascular) function.

To assess whether the microvasculature would also benefit from an alternate dietary regimen, we next analyzed kidney integrity.

### Alternate dietary regimen reduces renal inflammation

Renal mRNA expression levels of various genes related to endothelial activation (VCAM-1, ICAM-1, E-selectin, P-selectin) and inflammation (MCP-1, CD68) were significantly upregulated with HC feeding compared to CON. Strikingly, in the ALT group, these effects were either less pronounced (E-selectin, CD68) or even absent (VCAM-1, ICAM-1, P-selectin, MCP-1) (Table 2). MC diet feeding also had a stimulating effect on VCAM-1, ICAM-1 and E-selectin (moderate) but a strong upregulating effect (comparable to HC) on P-selectin, MCP-1, CD68.

**Table 2:** Renal mRNA expression.

	CON (n = 15)	HC (n = 13)	ALT (n = 13)	MC (n = 14)
VCAM-1	1.00±0.10^a^	1.39±0.14^b^	1.02±0.07^a^	1.10±0.11^ab^
ICAM-1	1.00±0.06^a^	1.51±0.20^b^	0.91±0.07^a^	1.05±0.07^a^
E-selectin	1.00±0.18^a^	1.85±0.27^b^	1.23±0.15^a^	1.39±0.16^ab^
P-selectin	1.00±0.11^a^	1.69±0.23^b^	1.14±0.14^ac^	1.57±0.21^bc^
MCP-1	1.00±0.11^ab^	1.33±0.15^a^	0.83±0.10^b^	1.32±0.15^a^
CD68	1.00±0.07^a^	1.53±0.10^b^	1.07±0.07^a^	1.51±0.10^b^

Renal mRNA expression of endothelial activation related genes (VCAM-1, ICAM-1, E-selectin, P-selectin) and inflammation related genes (MCP-1 and CD-68) relative to expression of CON group. Groups are abbreviated as: control mice fed a cholesterol-free Western type diet (CON); mice fed the same Western type diet but supplemented with high dose (1% w/w) cholesterol (HC); mice with alternate CON (4 days) and HC (3 days) diet regimen (ALT); mice fed Western type diet supplemented with a medium dose (0.43% w/w) cholesterol (MC). Data are expressed as means ± SEM. Values in the same row with different superscript letters are significantly different. P<0.05.

**Figure 4:**
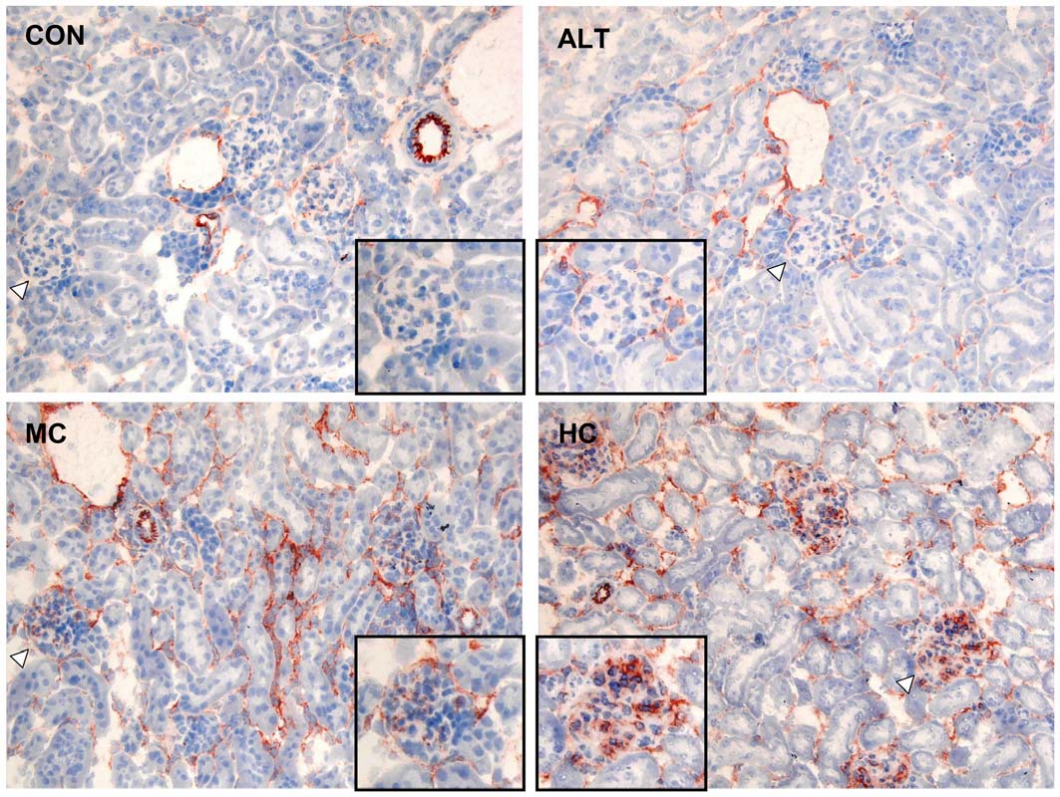
Immunohistochemistry of VCAM-1 in the kidney. A) Expression and localization of VCAM-1 in renal sections of each group. Insert shows a 200× magnification of a representative glomerulus which indicated by arrow. Control mice fed a cholesterol-free Western type diet (CON); high dose (1% w/w) cholesterol diet (HC); alternate CON (4 days) and HC (3 days) diet regimen (ALT); medium dose (0.43% w/w) cholesterol diet (MC).

Consistent with these gene expression findings, immunohistochemical analysis revealed an increased VCAM-1 immunoreactivity (IR) in glomeruli of HC treated mice relative to CON mice which showed hardly any IR. Under ALT diet conditions, glomerular VCAM-1 expression was not stimulated and remained comparably low as in CON. By contrast, chronic cholesterol exposure in the MC group was again associated with elevated VCAM-1 IR (Figure 4).

**Figure 5:**
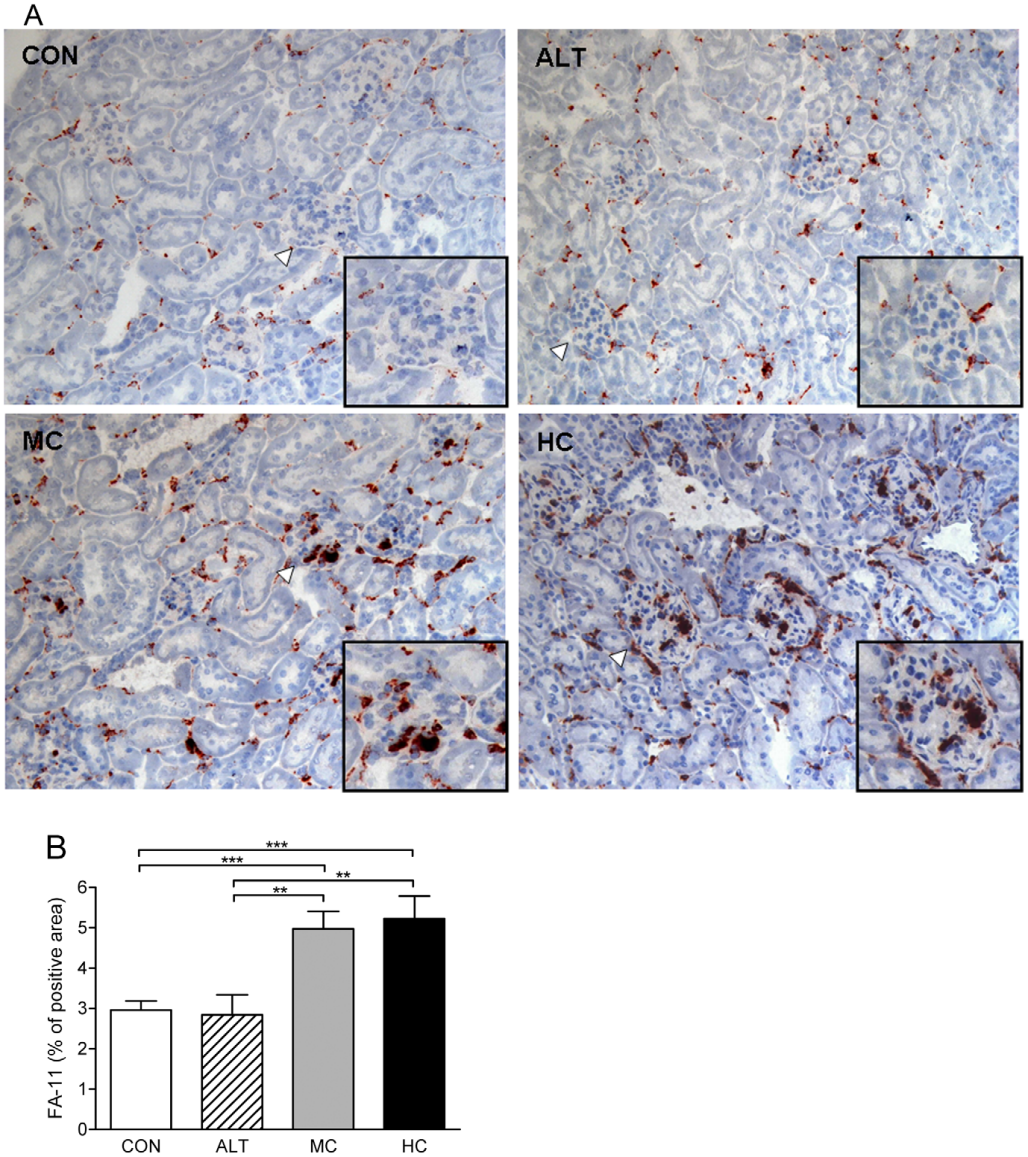
Immunohistochemistry for macrophages (CD68) in the kidney. A) Expression and localization of macrophages in renal sections. Inset shows a 200× magnification of a representative glomerulus which indicated by arrow. B) Renal accumulation of macrophages was quantified by morphometry. Data are expressed as a percentage of positive area occupied by FA-11 stained macrophages. Control mice fed a cholesterol-free Western type diet (CON, n = 15); high dose (1% w/w) cholesterol diet (HC, n = 13); alternate CON (4 days) and HC (3 days) diet regimen (ALT, n = 13); medium dose (0.43% w/w) cholesterol diet (MC, n = 14). Values are presented as means ± SEM. ** P<0.01, *** P<0.001.

Compared to CON animals, mice fed HC or MC showed significantly increased numbers of macrophages in glomeruli and tubulo-interstitium (Figure 5A) as quantified by morphometry (Figure 5B). Kidneys from ALT mice did not display any macrophage accumulation and were indistinguishable from CON mice.

To determine whether HC feeding would affect renal function, we measured urinary albumin and creatinine levels in spot urine samples. In all groups, urinary albumin/creatinine ratios were similar (data not shown) and within the normal range suggesting that the experimental diets had not yet affected the filtration function of the kidneys and that the present data reflect an early stage of renal disease. Consistent with this, analysis of Periodic acid-Schiff (PAS) stained renal cross-sections by light microscopy showed no overt renal abnormalities in all groups, except for a mild glomerular hypercellularity in mice that had been exposed to cholesterol chronically (HC and MC) (Figure S3).

## Discussion

In this study, we used a well documented, humanized mouse model of atherosclerosis, the ApoE*3Leiden mouse, to provide evidence that alternating high cholesterol (HC) -cholesterol-free (CON) diet can effectively diminish cardiovascular risk factors as compared with daily HC diet. The alternating feeding regimen reproduced most of the beneficial effects of daily cholesterol-free diet, including strongly diminished occurrence of hepatic, vascular and renal activation and inflammation. Furthermore, atherosclerosis in the ALT group was reduced by half compared to that in the HC group, albeit still higher than that in the CON group. By demonstrating that the concept of dietary manipulation is not restricted to caloric restriction but is also applicable to alternating dietary composition, our findings further underline the potential efficacy of the concept of alternate diet regimens. The perception that alternate cholesterol-free feeding (this paper) and alternate-day fasting [14]; [15] are almost as effective as daily cholesterol-free diet or daily caloric restriction respectively is of great societal importance. It may be anticipated namely that alternating-diet regimens may allow better compliance than would daily healthy diet regimens.

Although the efficacy of the alternate concept is intriguing, the exact molecular mechanisms underlying its favorable effects remain to be established and are an attractive area for further investigation. Some clues regarding the alternate HC diet might come from a previous study that we performed on the effects of dietary cholesterol on cardiovascular risk in ApoE3Leiden mice [16]. We showed that with increasing dietary cholesterol intake, plasma cholesterol levels increase and the liver switches from a resilient, adaptive state to an inflammatory, pro-atherosclerotic state, which is associated with an increase in endothelial dysfunction markers and early atherosclerotic lesion formation. For example, liver-derived inflammation markers such as serum amyloid A (SAA) and vascular activation markers such as soluble E-selectin, VCAM, and von Willebrand factor increase rapidly (within days) after consumption of a HC diet, and thus by far precede the onset of early aortic lesion formation. In the present study, the effects of alternating HC-CON diet were close to daily CON diet and much more favorable compared to daily 0.43% (w/w) cholesterol intake, despite an equal overall dietary cholesterol intake for the two groups. The elevated hepatic free cholesterol and NF-κB activity observed after feeding daily 1% or 0.43% dietary cholesterol (HC and MC), was not seen in the ALT or CON groups.

Similarly, we observed protective effects of ALT diet on the renal microvasculature. Endothelial adhesion molecules, including E-selectin, P-selectin, VCAM-1 and ICAM-1 were significantly upregulated in the HC group and moderately expressed in the MC group. In contrast, these diet-induced renal effects were markedly diminished in the ALT group displaying similar levels of expression as CON. Moreover, enhanced expression of MCP-1 in conjunction with significant renal macrophage infiltrates, particularly in the glomeruli, was observed in the HC and MC groups but was absent in the ALT group. These observations are in line with previous studies employing calorie restriction diets showing significant reductions in glomerular VCAM expression [17], renal monocyte/macrophage infiltration, mesangial expansion [18], and development of proteinuria [19].

Our results suggest that the daily dietary cholesterol intake of the HC and MC groups overcharges both hepatic and renal resilience, which results in a metabolic overload. This induces metabolic stress and inflammation [20] that equally affects both the macro (atherosclerosis) and microvasculature (liver and kidney). The ALT diet apparently provides these organs (liver and kidney) with a certain degree of elasticity which allows the mice to recover -during the cholesterol-free periods- from the intermittent periods of HC feeding, and thus to cope with alternating cholesterol stress.

Together, our own findings as well as the still nascent literature suggest that the principle of alternating dietary regimens can effectively modulate metabolic and functional risk factors. This approach would be a potentially attractive strategy to be tested for implementing more broadly in humans. If successful, it could provide a novel and more acceptable strategy to protect against the adverse effects of an unhealthy diet typical for the nutritional milieu which modern humans have created for themselves and as such could contribute to improve metabolic health. More work needs to be done to fully establish the concept and to address some of the outstanding questions. It needs to be ascertained whether the concept can also be extended to other alternating-diet regimens, including alternating saturated/unsaturated fat, alternating high/low-fat or alternating high/low-carbohydrate. An important issue also is whether the beneficial effects are valid both for disease prevention and delaying and/or improving existing metabolic diseases.

## Supporting Information

Figure S1 **Schematic illustration of feeding regimens.** Illustration of the feeding regimens during the experimental period. The red droplets indicate blood sampling time points. Experimental groups include from top to bottom; Control mice fed a cholesterol-free Western type diet (CON); high dose (1% w/w) cholesterol diet (HC); alternate CON (4 days) and HC (3 days) diet regimen (ALT); medium dose (0.43% w/w) cholesterol diet (MC).
(TIF)

Figure S2 **Representative photomicrographs of aortic root.** Photomicrographs after histological staining with hematoxylin-phloxine-saffron. Control mice fed a cholesterol-free Western type diet (CON); high dose (1% w/w) cholesterol diet (HC); alternate CON (4 days) and HC (3 days) diet regimen (ALT); medium dose (0.43% w/w) cholesterol diet (MC).
(TIF)

Figure S3 **Representative photomicrographs of kidney morphology.** Photomicrographs after histological PAS staining. Control mice fed a cholesterol-free Western type diet (CON); high dose (1% w/w) cholesterol diet (HC); alternate CON (4 days) and HC (3 days) diet regimen (ALT); medium dose (0.43% w/w) cholesterol diet (MC).
(TIF)
