# Supplementary figures and images for: Beneficial Effects of Alternate Dietary Regimen on Liver Inflammation, Atherosclerosis and Renal Activation
Source: PLoS One. 2011 Mar 31;6(3):e18432. doi: 10.1371/journal.pone.0018432 (PMC3069095; doi:10.1371/journal.pone.0018432)

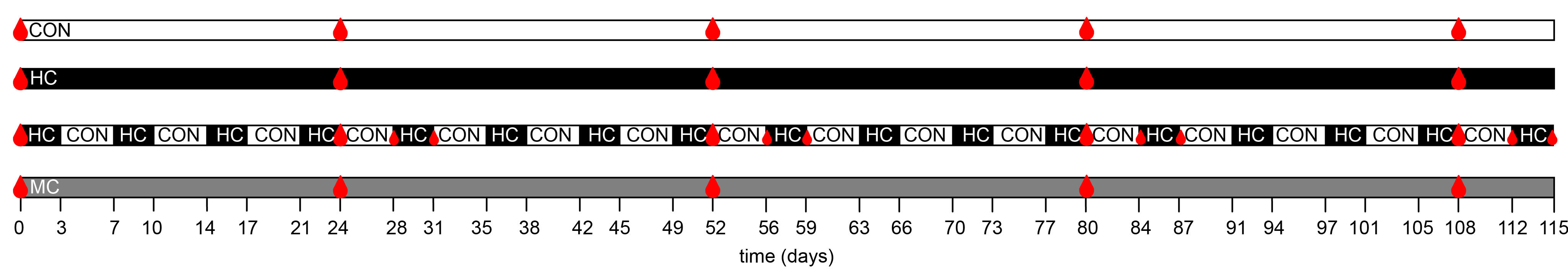

Supplement: Figure S1 — Schematic illustration of feeding regimens. Illustration of the feeding regimens during the experimental period. The red droplets indicate blood sampling time points. Experimental groups include from top to bottom; Control mice fed a cholesterol-free Western type diet (CON); high dose (1% w/w) cholesterol diet (HC); alternate CON (4 days) and HC (3 days) diet regimen (ALT); medium dose (0.43% w/w) cholesterol diet (MC). (TIF) [file pone.0018432.s001.tif]

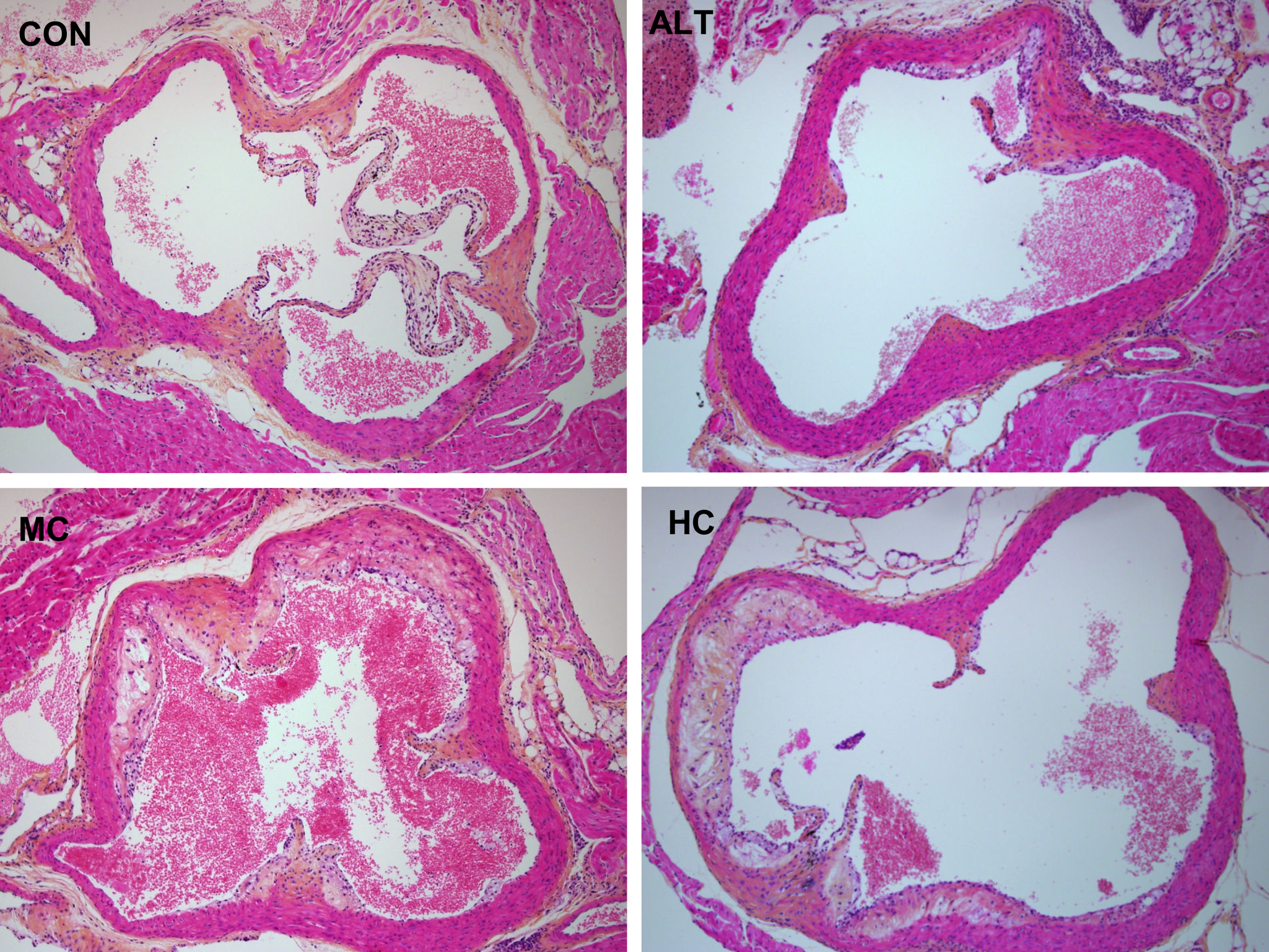

Supplement: Figure S2 — Representative photomicrographs of aortic root. Photomicrographs after histological staining with hematoxylin-phloxine-saffron. Control mice fed a cholesterol-free Western type diet (CON); high dose (1% w/w) cholesterol diet (HC); alternate CON (4 days) and HC (3 days) diet regimen (ALT); medium dose (0.43% w/w) cholesterol diet (MC). (TIF) [file pone.0018432.s002.tif]

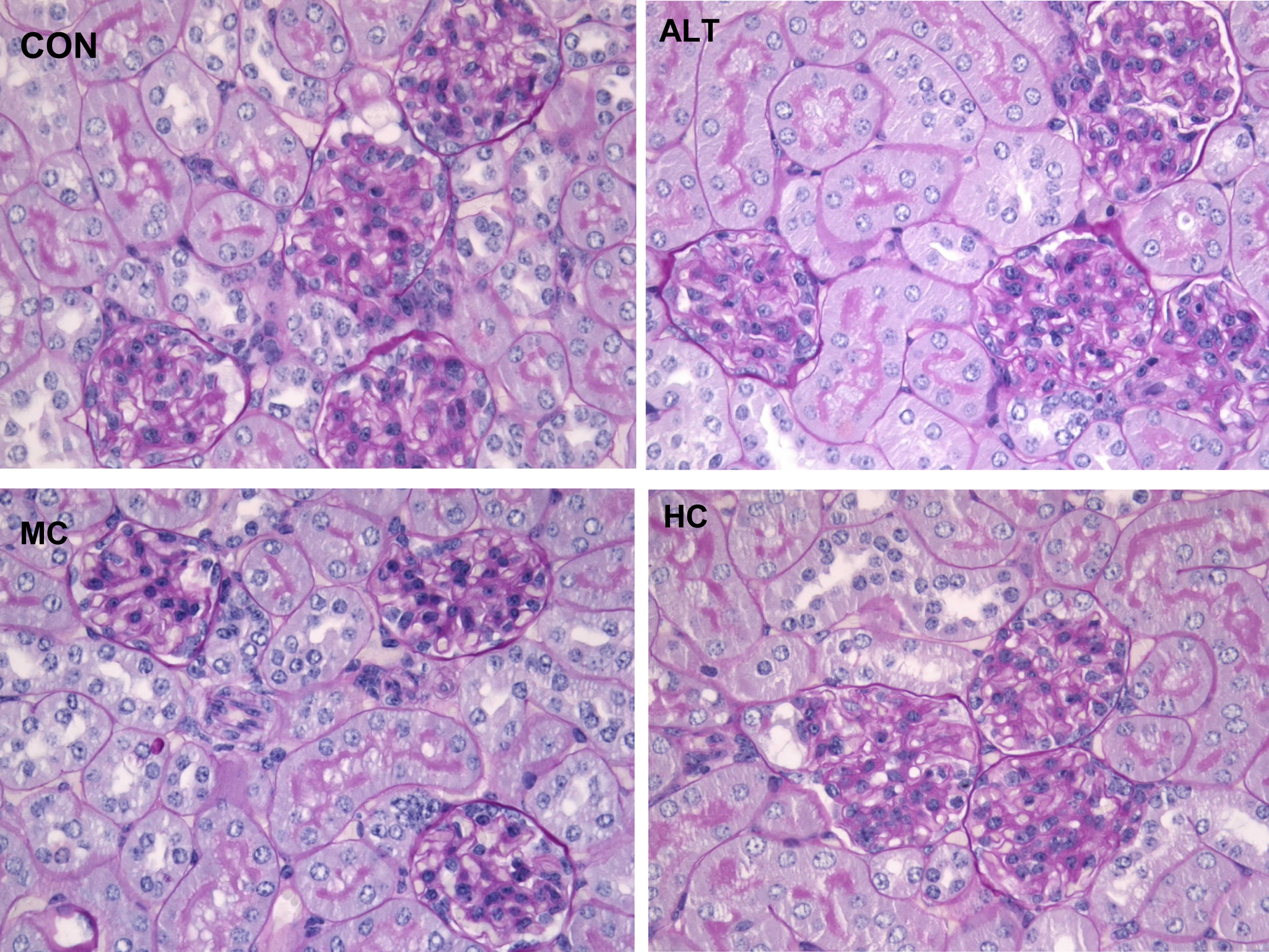

Supplement: Figure S3 — Representative photomicrographs of kidney morphology. Photomicrographs after histological PAS staining. Control mice fed a cholesterol-free Western type diet (CON); high dose (1% w/w) cholesterol diet (HC); alternate CON (4 days) and HC (3 days) diet regimen (ALT); medium dose (0.43% w/w) cholesterol diet (MC). (TIF) [file pone.0018432.s003.tif]
